# The Role of Insulin/IGF-1/PI3K/Akt/GSK3β Signaling in Parkinson's Disease Dementia

**DOI:** 10.3389/fnins.2018.00073

**Published:** 2018-02-20

**Authors:** Liying Yang, Hongyan Wang, Lijun Liu, Anmu Xie

**Affiliations:** ^1^Department of Neurology, Affiliated Hospital of Qingdao University, Qingdao, China; ^2^Department of Neurology, Qingdao Municipal Hospital, Qingdao, China

**Keywords:** Parkinson's disease dementia (PDD), insulin, Insulin-like growth factor (IGF-I), phosphoinositide 3 kinase (PI3k), Akt, Glycogen synthase kinase β (GSK3β), tau, α-synuclein

## Abstract

Dementia, a condition that frequently afflicts patients in advanced stages of Parkinson's disease (PD), results in decreased quality of life and survival time. Nevertheless, the pathological mechanisms underlying Parkinson's disease dementia (PDD) are not completely understood. The symptoms characteristic of PDD may be the result of functional and structural deficiencies. The present study implicates the accumulation of Lewy bodies in the cortex and limbic system as a potent trigger in the development of PDD. In addition, significant Alzheimer-type pathologies, including amyloid-β (Aβ) plaques and NFTs, are observed in almost half of PDD patients. Interestingly, links between PDD pathogenesis and the mechanisms underlying the development of insulin resistance have begun to emerge. Furthermore, previous studies have demonstrated that insulin treatment reduces amyloid plaques in Alzheimer's disease (AD), and normalizes the production and functionality of dopamine and ameliorates motor impairments in 6-OHDA-induced rat PD models. GSK3β, a downstream substrate of PI3K/Akt signaling following induction by insulin and IGF-1, exerts an influence on AD and PD physiopathology. The genetic overexpression of GSK3β in cortex and hippocampus results in signs of neurodegeneration and spatial learning deficits in *in vivo* models (Lucas et al., 2001), whereas its inhibition results in improvements in cognitive impairment in these rodents, including AD and PD. Accordingly, insulin- or IGF-1-activated PI3K/Akt/GSK3β signaling may be involved in PDD pathogenesis, at least in the pathology of PD-type + AD-type.

## Introduction

Parkinson's disease (PD), a major neurodegenerative disorder, is caused by dopaminergic neuronal loss in the substantia nigra as well as the formation of intracellular inclusion bodies, also known as Lewy bodies. In addition to the classic motor dysfunction symptoms of PD, non-movement disorders involving cognitive deficits and dementia are increasingly acknowledged as core symptoms of PD. Parkinson's disease dementia (PDD) has been reported across the entire course of PD but is particularly prevalent in advanced stages, resulting in high morbidity and mortality in approximately 80–90% by the age of 90 (Gratwicke et al., 2015).

Nevertheless, the underlying mechanisms of PDD pathogenesis have not been completely understood. Lewy bodies, neurofibrillary tangles (NFTs) and senile plaques may all contribute to the heterogeneous cellular pathology observed in cases of PDD (Wang et al., 2010). Interestingly, one of the most important recent findings is the link between PDD pathogenesis and the mechanisms underlying the development of insulin resistance; studies have found that patients with PDD are prone to comorbid insulin resistance (Bosco et al., 2012; Ashraghi et al., 2016), even when they were unaffected by Diabetes Mellitus. Furthermore, it has proven that Diabetes Mellitus increases the risk of developing Alzheimer's disease (AD). Given the high prevalence of dementia in advanced stages of PD, the impairment of multiple molecular cascades, such as insulin signaling, may amplify the neuropathological processes that lead to dementia before the overt manifestation of dementia. This review provides a summary of the data suggesting that insulin/insulin-like growth factor-1 (IGF-1) signaling and its downstream phosphatidylinositol 3-kinase (PI3K)/Akt/glycogen synthase kinase-3β (GSK-3β) cascades may be associated with the pathological processes of PDD.

## Clinical characteristics and pathophysiology of PDD

PDD clinically manifests as executive dysfunction, faulty recognition memory, visual hallucinations and cognitive fluctuations. Notably, the manifestation of executive dysfunction emerges at the early stage of or even prior to the onset of PD motor symptoms. The characteristics of PDD may result from functional deficiency, including a reduction in dopaminergic, cholinergic and other non-dopaminergic neurotransmitters, and from structural deficiency, including hippocampal and cortical atrophy, especially of the posterior occipital cortices (Bohnen et al., 2003; Emre, 2003).

Alpha-synuclein accumulation is associated with significant neuronal dysfunction. Compared with non-demented PD, PDD presents a distinct increase in α-synuclein pathology and a significant reduction in dopaminergic and cholinergic activity in cortical and hippocampal neurons (Hall et al., 2014). Reduced cortical volume or thickness in several regions, particularly the hippocampus, appears to be associated with progression to mild cognitive impairment and dementia. Recent study has suggested that a major trigger of the development of PDD is the accumulation of Lewy bodies in the cortex and limbic system (Irwin et al., 2013). Furthermore, significant Alzheimer-type pathologies, including amyloid-β (Aβ) plaques and NFTs, are also observed in patients with PDD (Irwin et al., 2013; Compta et al., 2014). Nevertheless, whether PDD patients involving AD-type pathology are also attacked by insulin resistance has not been referred in literatures. That may be due to the different emphasis of the authors' clarifying. Another study indicated that decreased Aβ and increased tau in the cerebrospinal fluid were predictors of PDD. The most prominent pathological feature of PD, Lewy body formation, is also detected in cases of advanced AD. Moreover, PD-type and AD-type pathologies often present in combination, accelerating and aggravating the progress of the disease, and suggesting a shared mechanism between AD and PDD pathogenesis. Insulin signaling pathways may be involved in this aspect of PDD pathology (PD-type pathology + AD-type pathology), and may influence their synergistic appearance.

## Insulin/IGF-1 signaling in PD and cognition impairment

Recently, impairment of insulin signaling has been found to increase the risk of AD (Craft et al., 2012; Talbot et al., 2012; Hölscher, 2014) and PD (Morris et al., 2008; Bosco et al., 2012; Ashraghi et al., 2016; Pang et al., 2016). A case control study illustrated that PDD patients had a higher prevalence of abnormal glucose metabolism, mainly insulin resistance, than non-demented PD patients (Bosco et al., 2012). Another study found that patients with PD and comorbid Diabetes Mellitus exhibited increased impairment in attentional function and executive function deficits than PD patients without Diabetes Mellitus (Ashraghi et al., 2016). These studies indicate that the impairment of insulin signaling may play a role in PD pathogenesis and could initiate or accelerate the development of PDD. Insulin is a peptide hormone secreted from the pancreas and may also be synthesized in the brain. Insulin crosses the blood–brain barrier and is internalized by neurons to activate several signaling pathways via binding to insulin receptor (IR) and activating insulin receptor substrate-1 (IRS-1). IR is expressed by glial cells and neurons, including olfactory bulb, cerebral cortex, hypothalamus and hippocampus. Through binding with IR, insulin affects glycometabolism and food intake. In the central nervous system, insulin plays a role in learning and memory processes and is associated with hippocampal long-term potentiation (LTP) facilitated by insulin (Lee et al., 2005). Insulin treatment has been shown to have a positive effect on nervous system development and growth, and can alleviate and repair damage caused by the inflammation response. Previous studies have suggested that insulin treatment has an effect on reducing amyloid plaques in patients with AD, normalizing dopamine production and functionality and ameliorating motor impairments in the 6-OHDA-induced rat PD model (Hölscher, 2014; Pang et al., 2016). Nasal application of insulin could improve memory in patients with AD (Craft et al., 2012). However, whether insulin therapy improves cognitive function or dementia in PD patients has not yet been assessed.

The neurogrowth and neurotrophic factor IGF-1, a 70 amino acid protein, has been confirmed to exert a neuroprotective and proliferative function through the promotion of cell survival, prevention of apoptosis, and stimulation of neurogenesis in regions such as the hippocampus. IGF-1 crosses the blood brain barrier and may also be produced by neurons and glial cells. IGF-1 is internalized by binding to IGF-1 receptor (IGF-1R), which is widely expressed by neurons, to activate IRS-2. It has been demonstrated that IGF-1 exerts a positive effect on dopaminergic neurons in both *in vitro* and *in vivo* models of PD (Offen et al., 2001; Kao, 2009). Westwood et al. (2014) found that lower serum IGF-1 levels were associated with increased risk of AD dementia as well as increased risk of all forms of dementia, including PDD, after adjustment for several other known risk factors. In addition, another report suggested that lower serum IGF-1 levels were associated with cognitive function in patients with PD (Ma et al., 2015).

**Figure 1:**
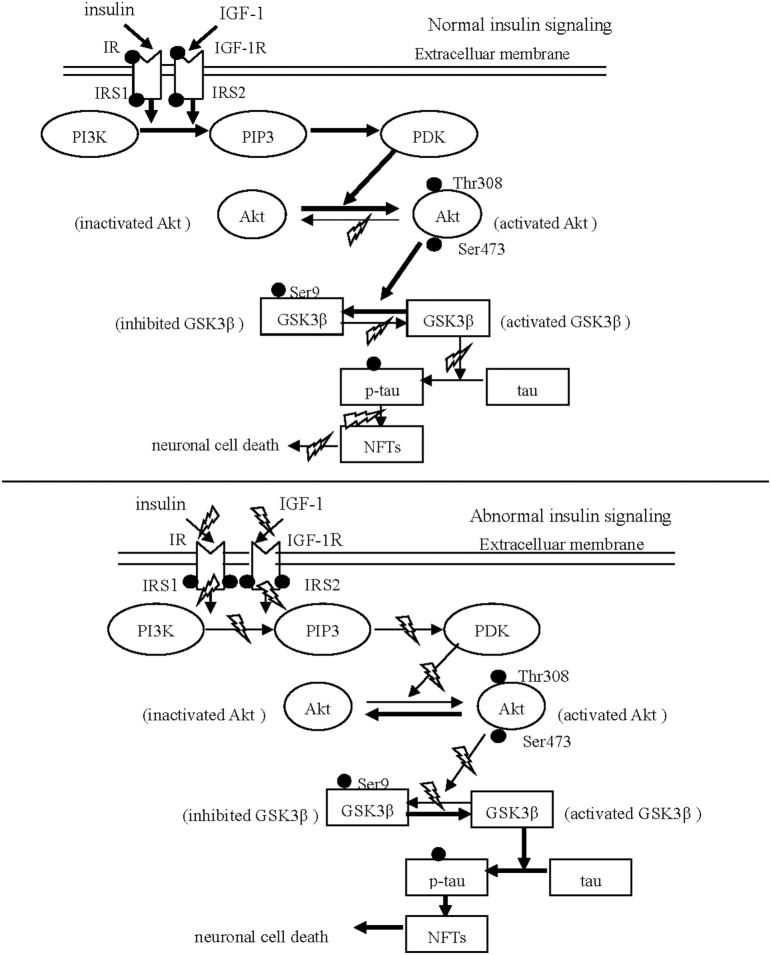
Schematic drawing of insulin/IGF-1/PI3K/Akt/GSK3β/tau signaling. The normal insulin signaling pathway Insulin and IGF-1 are internalized neurons via respectively binding to IR and IGF-1 R. After phosphorylation of IR and IGF-1 R, IRS is phosphorylated to initiate the down-stream substrate, PI3K. Subsequently, Akt is activated in response to PI3K signaling via phosphorylated by PDK. The activated Akt phosphorylates GSK31β at Ser 9, leading to GSK31β inhibited. The abnormal insulin signaling pathway Insulin and IGF-1 can not trigger the down-stream signaling in PD and AD, resulting from the reduced phosphorylation of IR and IGF-1 R and from the increased hyper-phosphorylation of IRS-1 and IRS-2. The inhibition of Akt activates GSK3β through reducing GSK3β phosphorylation at serine 9. Subsequently, activated GSK3β stimulates aberrant tau phosphorylation, and neuronal death.

Insulin and IGF-1 share similar downstream signaling pathways leading to several shared outcomes, such as activation of the PI3K/Akt pathway after IRS-1 and IRS-2 phosphorylation. Recent histological and biochemical analyses have revealed that the reduced phosphorylation of IR and the increased hyper-phosphorylation of IRS, the inactivated proteins, were observed in both AD and PD patients (Morris et al., 2008; Talbot et al., 2012). This results in the failure of insulin and IGF-1 to activate downstream molecules such as PI3K. In contrast, when the IRS is properly phosphorylated, insulin and IGF-1 result in a decrease in the neurotoxicity and accumulation of α-synuclein by triggering downstream PI3K/Akt signaling (Kao, 2009). Wang et al. (2010) reported that IGF-1 protected cells of the S-type human neuroblastoma line (SH-EP1) from MPP+-induced apoptosis through the cytoprotective PI3K/Akt signaling pathway, which required GSK3β inactivation. Generally, the activation of components of the insulin/IGF-1 signaling pathway occurs in the following sequence: insulin or IGF-1 → IR or IGF-1R → IRS-1/2 → PI3K → Akt → GSK3β (see Figure 1).

## PI3K/Akt signaling in PD and cognition impairment

The PI3K/Akt signaling pathway, a key molecular signal transduction pathway, has been linked to several diseases, including Diabetes Mellitus and cancer by means of its ability to regulate biochemical and cellular pathological processes involving glycolysis, the cell cycle and apoptosis. This pathway is also important for regulating neuronal survival and synaptic plasticity during aging and many neurodegenerative diseases. PI3K, which is activated by IRS, triggers activation of the downstream protein phosphatidylinositol 3, 4, 5-triphosphate (PIP3). PIP3 then activates phosphoinositide-dependent protein kinase (PDK), which subsequently recruits Akt (also termed protein kinase B, PKB) to the plasma membrane and phosphorylates Akt on residues Thr 308 and Ser 473. Majd et al. (2013) have reported that the pathological features of PD and AD—amyloid senile plaques and Lewy bodies—frequently coexist in regions of the neocortex and hippocampus, which are associated with dementia. PI3K/Akt-mediated pathways are involved in several neurodegenerative diseases, including AD and PD, and may contribute to the coexistence of Aβ and α-synuclein in PDD. Furthermore, it was reported that 6-OHDA- and MPP+-induced neurodegeneration was associated with GSK3β in *in vitro* PD models (Wu et al., 2007), further strengthening the link between GSK3β and neuronal death.

Accordingly, the activated PI3K/Akt signaling fosters endothelial survival, limits neuronal injury, and blocks inflammatory neuron death during the pathophysiological process of PD. For example, the ratio of phospho-Akt/total-Akt decreases in dopaminergic neurons (Malagelada et al., 2008), supporting the notion that Akt-mediated signaling pathways are suppressed in PD pathogenesis. Moreover, Akt participates in the modulation of dopaminergic transporters. The following mechanisms associated with Akt contribute to cellular survival: (1) Akt regulates the pro-apoptosis and anti-apoptosis balance by inhibiting pro-apoptotic cytokines while activating anti-apoptotic cytokines, thereby influencing neuronal survival. (2) PI3K/Akt, which is induced by insulin or IGF-1, plays a major role in the regulation of autophagy, protein synthesis and actin cytoskeleton via the Mammalian target of rapamycin (mTOR) signal activation to directly or indirectly promote neuronal survival (Lee et al., 2005). (3) Through increasing or decreasing GSK3β phosphorylation at serine 9, Akt also performs a crucial negative regulatory function on GSK3β, thereby affecting synaptic plasticity and neuronal survival. For example, inhibition of Akt activates GSK3β through reducing GSK3β phosphorylation at serine 9, whereas activation of Akt inhibits GSK3β through enhancing phosphorylation. Accordingly, the negative regulation of GSK3β by Akt exerts a crucial function in cell cycle progression and metabolism in neurodegenerative disorders, including PD (Wang et al., 2007) and AD (Balaraman et al., 2006).

## GSK3β in PD and cognition impairment

The GSK3 proteins, GSK3α and GSK3β, is expressed in all eukaryotic cells, including neurons and neuroglia, and is thought to be primarily involved in glycogen synthesis. In addition to its known involvement of glycometabolism, GSK3β is involved in neuronal growth, neuro-proliferation, differentiation and apoptosis in neurodegenerative diseases. Furthermore, GSK3β performs a crucial function in α-synuclein-mediated neurodegeneration. The overexpression of GSK3β in cortex and hippocampus results in signs of neurodegeneration and spatial learning deficits in *in vivo* models (Lucas et al., 2001), whereas its inhibition results in improvements in cognitive impairment in these rodents. Furthermore, King and colleagues (King et al., 2014) reviewed evidence that lithium treatment, which inhibited GSK3, could alleviate spatial memory impairment in the Morris water maze test in a wide variety of animal models of central nervous system diseases, including AD and PD.

Ser 9 is the site of GSK3β inhibition, while Tyr 216 is the site of GSK3β activation. Thus, phosphorylation of GSK3β at the Ser 9 results in inhibition of GSK3β, while the de-phosphorylation of this site results in activated GSK3β. The PI3K/Akt signaling, which is activated by insulin or IGF-1, negatively regulates GSK3β through increasing or decreasing phosphorylation at Ser 9. It has been proven that insulin effectively increases the phosphorylation GSK3β at Ser 9, thereby inactivating GSK3β, via up-regulating PI3K/Akt signaling (Wang et al., 2013). Meanwhile, Deng et al. (2005) have found that the use of a PI3K inhibitor increased tau hyperphosphorylation via GSK activation. Accordingly, the selective downregulation of PI3K/Akt signaling, combined with the elevation of GSK-3β activity, could trigger or aggravate the onset of dysfunctional brain pathogeneses such as PDD. Several mechanisms associated with GSK3β appear to contribute to cognitive impairment, as detailed below.

## LTP and long-term depression (LTD)

Two major forms of synaptic plasticity—LTP and LTD—are used to receive and store information in hippocampus. The imbalance of LTP and LTD in hippocampus results in synaptic dysfunction and memory disorders. Given that hippocampal synaptic plasticity participates in learning and memory formation, synaptic dysfunction is known to be linked to AD. Similar to AD, PDD also presents with hippocampal atrophy and neuronal death, suggesting that synaptic dysfunction is also involved in the pathogenesis of dementia in PD patients.

Phosphorylated GSK3β improves long-term memory in hippocampal-associated tasks. During LTP, the activity of GSK3β is reduced through phosphorylation at Ser 9 (resulting in inhibited GSK3β), which is mediated by PI3K/Akt signaling (Hooper et al., 2007; Peineau et al., 2007). During LTD, in contrast, the activity of GSK3β is indirectly enhanced through the reduced phosphorylation at Ser 9 (resulting in activated GSK3β) via Akt. (Peineau et al., 2007). In addition, genetic overexpression of GSK3β hampers the induction of LTP, while pharmacological inhibition of GSK3β obstructs the induction of N-methyl-D-aspartate (NMDA) receptor-dependent LTD. Via phosphorylation or dephosphorylation at Ser 9, GSK3β plays a vital role in regulating the balance of LTP and LTD, which subsequently affects learning and memory formation in PD.

## Tau, α-synuclein and Aβ

The neuropathological forms of α-synuclein, Aβ and tau, often coexist in the advanced stages of PD and AD. Close examination has revealed that tau hyper-phosphorylation is not a specific marker for AD, because these abnormal proteins have been detected in other neurodegenerative diseases, such as PD (Jellinger, 2009; Compta et al., 2014; Modreanu et al., 2017). Moreover, it was reported that almost half of PDD patients also developed amyloid plaques and tau-containing NFTs (Irwin et al., 2013), both of which are hallmarks of AD pathology. In comparison with pure PDD (defined by the presence of only Lewy bodies), AD-type PDD (PD-type pathology + AD-type pathology) is marked by more significant α-synuclein accumulation in the cortex and limbic regions (Kazmierczak et al., 2008). This suggests a potential pathological synergy between α-synuclein, Aβ and tau, in which GSK3β-mediated signaling pathways may be involved.

GSK3β is an important kinase known to hyper-phosphorylate tau through phosphorylation at Thr 23 (Guo et al., 2017). Hyper-phosphorylation of tau leads to its intracellular accumulation, which in turn leads to the formation of NFTs and the failure to bind and assemble microtubules. Consequently, the accumulation of abnormal tau disturbs synaptic function, hinders axonal transport, influences neurotrophic function, and ultimately results in neuronal death by triggering apoptotic signaling.

**Figure 2:**
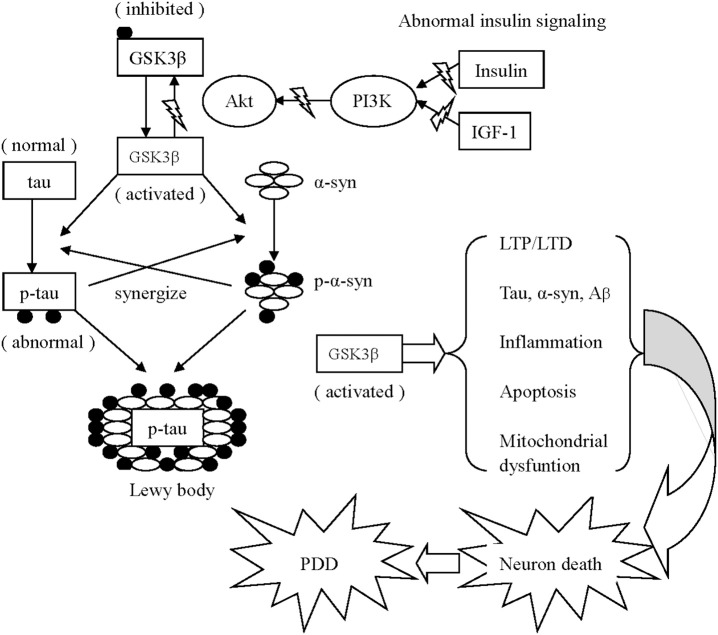
Schematic drawing of several mechanisms associated with GSK3β appear to contribute to PDD 1. Activated GSK3β is known to hyper-phosphorylate tau, which leads to intracellular accumulation and failure to bind and assemble microtubules. Meanwhile, α-synuclein phosphorylation is also enhanced via activated GSK3β. Additionally, the phosphorylated α-synuclein and tau enhance the phosphorylation of unphosphorylated α-synuclein and tau of each other to accelerate the progress of neurodegeneration. Similarly, α-synuclein could work in coordination with Aβ to increase cytotoxicity. Both α-synuclein and Aβ overexpression are associated with elevated levels of active GSK3β, resulting in increased tau hyper-phosphorylation. 2. Activated GSK3β impairs long-term memory in hippocampal-associated tasks via imbalanced LTP and LTD. 3. The GSK3β-related imbalance of pro-inflammation and anti-inflammation continuously aggravates PD pathology. 4. Activated GSK3β negatively effects mitochondrial function, elevates the expression of caspase-3 and caspase-9, and ultimately induces neuronal apoptosis.

Furthermore, abnormal tau and α-synuclein act synergistically to interfere with neuronal survival in neurodegenerative disorders. On one hand, low concentrations of α-synuclein do not fibrillate until tau is present (Giasson et al., 2003). In addition to promoting the synthesis of α-synuclein, tau increases the insoluble form and strengthens the toxicity of α-synuclein. On the other hand, α-synuclein enhances tau polymerization and accumulation in a GSK3β-dependent manner. The addition of α-synuclein activates GSK3β by reducing Ser 9 phosphorylation (thereby inhibiting GSK3β) and elevating Tyr 216 phosphorylation (thereby activating GSK3β), thereby contributing GSK3β-dependent tau hyper-phosphorylation (Gassowska et al., 2014) (see Figure 2). Conversely, GSK3β inhibition decreases the phosphorylation and accumulation of α-synuclein *in vitro* in PD pathology (Yuan et al., 2015).

Similarly to the cooperative effect of α-synuclein and tau, α-synuclein could work in coordination with Aβ to increase cytotoxicity. Aβ42 effectively promotes the oligomerization of α-synuclein *in vitro* (Masliah et al., 2001), while extracellularly applied α-synuclein increases the secretion of Aβ and potentiated its toxicity. Consequently, α-synuclein and Aβ reciprocally impair mitochondrial function and increase neuronal death (Yuan et al., 2015). Furthermore, Aβ exposure induces GSK3β activity, whereas GSK3 proteins (including GSK3α and GSK3β) both promote the production of Aβ and stimulate apoptotic signaling via Aβ. Both α-synuclein and Aβ overexpression are associated with elevated levels of active GSK3β, resulting in increased tau hyper-phosphorylation.

## Inflammation

A substantial number of *in vivo* and *in vitro* trials have revealed that neuroinflammatory responses contribute to the pathology of neurodegenerative disorders, including PD, Lewy body dementia and AD. In patients with PD, increased levels of pro-inflammatory cytokines, accompanied by anti-inflammatory cytokines, are detected in serum and cerebrospinal fluid, supporting the above notion. Furthermore, the overexpression and accumulation of α-synuclein in these neurons decreases dopamine biosynthesis, aggravates mitochondrial dysfunction, and directly activates glia to increase inflammatory stress, thereby contributing to synergistic neurodegeneration.

The activation of GSK3β, which is negatively regulated by upstream PI3K/Akt signaling, participates energetically in neuroinflammatory progression through activating glia. Activated GSK3β upregulates the NF-κB pathway (Zhang et al., 2014) to produce pro-inflammatory cytokines (such as TNF-α, IL-1β, and IL-6), while reducing the synthesis of anti-inflammatory factors such as IL-10 (Martin et al., 2005). The GSK3β-related imbalance of pro-inflammation and anti-inflammation continuously aggravates PD pathology.

## Apoptosis

Although most GSK3β protein is cytosolic, a small amount of GSK3β functions in mitochondria and the nucleus in patients with neurodegenerative diseases. Activated GSK3β negatively effects mitochondrial function, elevates the expression of caspase-3 and caspase-9, and ultimately induces neuronal apoptosis. In contrast, GSK3β inhibition reduces the synthesis of pro-caspase and caspase cytokines, thereby alleviating neuronal apoptosis. Interestingly, it has been proven that IGF-1 inhibits the JNK-mediated apoptotic pathway through inactivating GSK3β, an effect mediated by PI3K/Akt signaling (Wang et al., 2010). Moreover, aggregated α-synuclein, Aβ and hyper-phosphorylated tau directly induce apoptotic signaling, which is enhanced by activated GSK3β (Mines et al., 2011).

## Conclusions

Many factors lead to the heterogeneous pathology of PDD, including losses of function (i.e., dopaminergic and non-dopaminergic dysfunction) and structure (i.e., cortical and limbic atrophy). PD-type pathology combined with AD-type pathology is an important pathological subtype of PDD. PI3K/Akt/GSK3β signaling, which is triggered by insulin and IGF-1, may perform a significant role in the neuronal survival and pathological aggregation of tau, Aβ and α-synuclein. The mechanisms associated with GSK3β that contribute to cognitive impairment are as follows. First, GSK3β regulates the balance of LTP and LTD. Second, GSK3β hyper-phosphorylates tau and synergistically enhances the synthesis and neurocytotoxicity of tau, Aβ and α-synuclein. Finally, GSK3β activates inflammatory stress and apoptosis. In conclusion, insulin and IGF-1 may alleviate cognitive impairment in PD via the inactivation of GSK3β mediated by PI3K/Akt.

The underlying mechanisms of PDD pathogenesis are obscure and complex. There are multiple kinases and signaling pathways initiate different downstream substrates involving in the pathogenesis of PDD. Nevertheless, PDD pathology has much in common with other neurodegenerative diseases, including AD, Lewy body dementia and frontotemporal dementia. Hence, the pathogenesis of PDD cannot be elucidated by a single mechanism. This article summarizes previous literature suggesting that abnormalities in insulin signaling may be relevant to the pathological mechanism of PDD, in particular cases with AD-type pathology. Nevertheless, it has not been determined whether abnormal insulin signaling is the factor only responsible for the hallmarks of PDD pathology, including senile plaques and NFTs. Does insulin signaling perform a significant function in the pure Lewy body form of PDD? It is necessary to carry out retrospective and prospective cohort studies to identify the relationship between insulin resistance and the different pathological types of PDD. More importantly, the PI3K/Akt/GSK3β signaling cascade induced by insulin/IGF-I is worth additional examination in *in vitro* and *in vivo* trials, providing a new strategy for intervention in PDD patients.

## Author contributions

LY carried out electronic literature searches and drafted the manuscript. HW and LL participated in the study design. AX carried out reviewing the manuscripts. All authors read and approved the final manuscript.

### Conflict of interest statement

The authors declare that the research was conducted in the absence of any commercial or financial relationships that could be construed as a potential conflict of interest.
